# Unveiling the cholesterol-hemangioma axis: a path to new treatments

**DOI:** 10.1172/JCI193039

**Published:** 2025-04-01

**Authors:** M. Luisa Iruela-Arispe

Infantile hemangiomas are benign vascular tumors that typically appear in early life and continue to grow over several months in a proliferative phase. The beta blocker propranolol was identified as an effective treatment option for infantile hemangioma and is currently the first-line therapy considered for infants with severe or complicated disease (1). From the outset, propranolol’s mechanism of action on infantile hemangioma was assumed to be through its beta blocker activity (2), yet direct functional evidence has been missing. In this issue, the Bischoff laboratory clarified the mechanism of action and uncovered an important link to cholesterol metabolism (3).

Previous research from the Bischoff and Francois laboratories identified SOX18, an endothelial transcription factor, as a key target of propranolol therapy in infantile hemangioma (4). In the current study, the team set out to learn which genes were affected when SOX18 transcriptional activity was disrupted by R(+) propranolol, the enantiomer of propranolol that lacks beta blocker activity (3).

The investigators designed a simple RNA-Seq experiment in which they treated hemangioma stem cells with R(+) propranolol. To their surprise, they found that, at the onset of endothelial differentiation, R(+) propranolol treatment downregulated genes needed for cholesterol/isoprenoid biosynthesis — including the mRNA encoding the rate-limiting enzyme in the HMG-CoA-reductase pathway. The authors show that SOX18 is both necessary and sufficient for the R(+) propranolol–mediated reduction of the cholesterol/isoprenoid pathway — also known as the mevalonate pathway (MVP) — and implicate a coordinated action of SOX18 with the master transcriptional regulator SREBP2 (4), although the nature of this coordinated effort was not clarified in this study.

In addition, the unexpected connection between SOX18 and the MVP prompted the question of whether blood vessels in infantile hemangioma could be suppressed by statins, as these drugs are competitive inhibitors of HMG-CoA-reductase and well known for their contributions to cardiovascular health (5). The authors show that either one of two commonly prescribed statins, simvastatin or atorvastatin, inhibit the formation of human hemangioma blood vessels in a murine xenograft model (3).

There are several important implications of this study. The findings suggest that statins could potentially be repurposed to treat children with infantile hemangioma alone or in combination with propranolol. The two drugs would be acting on the same pathway: propranolol, via its R(+) enantiomer, at SOX18 and statins downstream at the MVP. Perhaps the two drugs could be effective at a lower dose when combined compared with either drug used alone at standard dose. This concept will require clinical studies, which could examine the potential for more complete therapeutic responses and/or shortened treatment, as current propranolol therapy is often a year or longer. Future work may also assess if the SOX18-MVP axis is operating in the endothelium of other types of vascular diseases such as vascular malformations or perhaps in tumor angiogenesis. Finally, clinical use of propranolol or statins is associated with improved outcomes for patients with cancer (5–7), potentially due to an antitumor angiogenesis effect, suggesting that further study of these drugs in cancer is also warranted.

## Version 1 04/01/2025

Electronic publication
